# Fermented Korean Red Ginseng Extract Enriched in Rd and Rg3 Protects against Non-Alcoholic Fatty Liver Disease through Regulation of mTORC1

**DOI:** 10.3390/nu11122963

**Published:** 2019-12-04

**Authors:** Su-Yeon Choi, Jeong-Su Park, Chang-Ho Shon, Chae-Young Lee, Jae-Myun Ryu, Dong-Ju Son, Bang-Yeon Hwang, Hwan-Soo Yoo, Young-Chang Cho, Jin Lee, Jong-Won Kim, Yoon-Seok Roh

**Affiliations:** 1 College of Pharmacy and Medical Research Center, Chungbuk National University, Cheongju 28160, Korea; yeonybly@gmail.com (S.-Y.C.); 6318js@gmail.com (J.-S.P.); ryan8840@gmail.com (C.-H.S.); lego0325@naver.com (C.-Y.L.); sondj1@chungbuk.ac.kr (D.-J.S.);; 2 NOVA K-MED Co., Ltd., 1646 Yuseong-daero, HNU Innobiz Park Suite 403, Yuseong-gu, Daejeon 34054, Korea; ryujm@novarex.co.kr; 3 College of Pharmacy, Chonnam National University, Gwangju 61186, Korea; 4 Department of Pathology, School of Medicine, University of California, San Diego, CA 92093, USA; jil327@ucsd.edu; 5 Biosafety Research Institute and College of Veterinary Medicine, Jeonbuk National University, Deokjin-gu, Jeonju-si 54596, Korea

**Keywords:** NAFLD, fermented Korean red ginseng, mTORC1

## Abstract

The fermentation of Korean red ginseng (RG) increases the bioavailability and efficacy of RG, which has a protective role in various diseases. However, the ginsenoside-specific molecular mechanism of the fermented RG with *Cordyceps militaris* (CRG) has not been elucidated in non-alcoholic fatty liver disease (NAFLD). A mouse model of NAFLD was induced by a fast-food diet (FFD) and treated with CRG (100 or 300 mg/kg) for the last 8 weeks. CRG-mediated signaling was assessed in the liver cells isolated from mice. CRG administration significantly reduced the FFD-induced steatosis, liver injury, and inflammation, indicating that CRG confers protective effects against NAFLD. Of note, an extract of CRG contains a significantly increased amount of ginsenosides (Rd and Rg3) after bioconversion compared with that of conventional RG. Moreover, in vitro treatment with Rd or Rg3 produced anti-steatotic effects in primary hepatocytes. Mechanistically, CRG protected palmitate-induced activation of mTORC1 and subsequent inhibition of mitophagy and PPARα signaling. Similar to that noted in hepatocytes, CRG exerted anti-inflammatory activity through mTORC1 inhibition-mediated M2 polarization. In conclusion, CRG inhibits lipid-mediated pathologic activation of mTORC1 in hepatocytes and macrophages, which in turn prevents NAFLD development. Thus, the administration of CRG may be an alternative for the prevention of NAFLD.

## 1. Introduction

Non-alcoholic fatty liver disease (NAFLD) is becoming one of the most common chronic liver diseases in the world, with a prevalence of 20%–30% of the adult population in western countries. The spectrum of NAFLD ranges from simple steatosis, non-alcoholic steatohepatitis (NASH), fibrosis, cirrhosis, and eventually hepatocellular carcinoma [1,2,3,4]. One of the features of NAFLD is fat accumulation in the liver, which increased the symptoms of metabolic syndrome characterized by obesity, dyslipidemia, and insulin resistance [5]. Although there are many studies on NAFLD, there is no approved drug for the treatment of NAFLD yet.

Mammalian target of rapamycin (mTOR) exists in two conserved protein complexes, mTORC1 and mTORC2. mTORC1 has been demonstrated to control cell growth and metabolism in response to nutrients, growth factors, cellular energy, and stress [6,7]. Especially, aberrant activation of mTORC1 has been suggested to be a critical contributor to the development of NAFLD [8]. For instance, excessive mTORC1 activity enhances lipogenesis by the induction of Sterol regulatory element-binding protein 1 (SREBP1c) [9,10,11,12]. Furthermore, mTORC1 activation suppresses peroxisome proliferator-activated receptor α (PPARα) activity, which in turn inhibits β-oxidation in the liver [13,14]. mTORC1 has been shown to suppress the autophagy pathway via regulation of autophagy activation kinase 1 (ULK1) [15,16]. Thus, control of aberrant activation of mTORC1 may be a target for the prevention of fat accumulation in the liver.

The Korean red ginseng (RG, the steamed root of *Panax ginseng* Meyer, family Araliaceae) is most frequently used in Asian countries for thousands of years and has been used as a nutritional supplement to improve health [17,18]. RG has protective effects against hyperglycemia, obesity, and free radical-induced oxidative stress [19,20,21]. The well-known major active constituents in RG are ginsenosides, a group of saponins with triterpenoid dammarane structure. It is known that orally ingested ginsenosides in RG pass through the stomach and small intestine without decomposition by either gastric juice or liver enzymes into the large intestine, where ginsenosides are metabolized to bioactive forms by intestinal bacterial deglycosylation and fatty acid esterification in the body [22,23,24]. Therefore, the deglycosylation process of ginsenosides is crucial for its biological activity. However, the oral bioavailability of intact ginsenosides from the intestines is low and varies from person to person [25,26]. An individual’s intestinal microflorae are very changeable depending on host conditions, including diet, health, and even stress. Therefore, the efficacy of transformation and bioavailability of ginsenosides may be partly associated with the intestinal microflora and differ greatly due to the diversity of resident microflora between individuals.

Many different strategies have been developed to improve the health-beneficial effect of RG by transforming ginsenosides into their aglycone forms. Several studies have shown that the transformation of ginsenosides into deglycosylated ginsenosides is required in order for them to enhance their biological activities [27]. Various methods have been suggested for transforming the chemical composition of RG using mild acid hydrolysis, enzymatic conversion, and microbial conversion via fermentation can improve the oral absorption and bioavailability of RG [28,29,30,31]. However, chemical methods produce side reactions such as epimerization, hydration, and hydroxylation, and most of the microorganisms used for the transformation of ginsenosides are not food-grade standards [32].

The purpose of this study was to enhance the health-beneficial properties of RG by using solid-state fermentation with *Cordyceps militaris*, a medicinal and edible mushroom. The medicinal caterpillar fungus *C. militaris* is the only cultivated caterpillar fungus whose fruiting bodies can be formed without the process of caterpillar infection. It contains numbers of bioactive constituents, including adenosine, cordycepin, and polysaccharides [33]. Currently, cultivation methods of *C. militaris* mainly include solid-state fermentation, submerged fermentation, and membrane-surface liquid cultivation [34]. Furthermore, it has been shown that the solid-state fermentation of grains by *C. militaris* results in biotransformation grains with high antioxidant activity, DNA damage protection, and angiotensin I-converting enzyme inhibitory activity, thereby providing a method to obtain grains with enhanced bioactive properties [35,36].

Although studies have shown that RG mitigates NAFLD by inhibiting the inflammatory response [37], the mechanistic role of fermented RG enriched in ginsenosides remains poorly understood. Therefore, in the present study, solid-fermentation of RG by *C. militaris* was studied to find a technological method to potentiate bioactive properties of RG against NAFLD and its mechanism of action. In the current study, we found the Rd and Rg3-enriched extract of *C. militaris*-fermented RG with *Cordyceps militaris* (CRG) ameliorates NAFLD through mTORC1 inhibition-mediated mitophagy induction in hepatocytes and M2 polarization in macrophages, respectively.

## 2. Materials and Methods

### 2.1. Preparation for CRG Extract

**Scheme 1:**
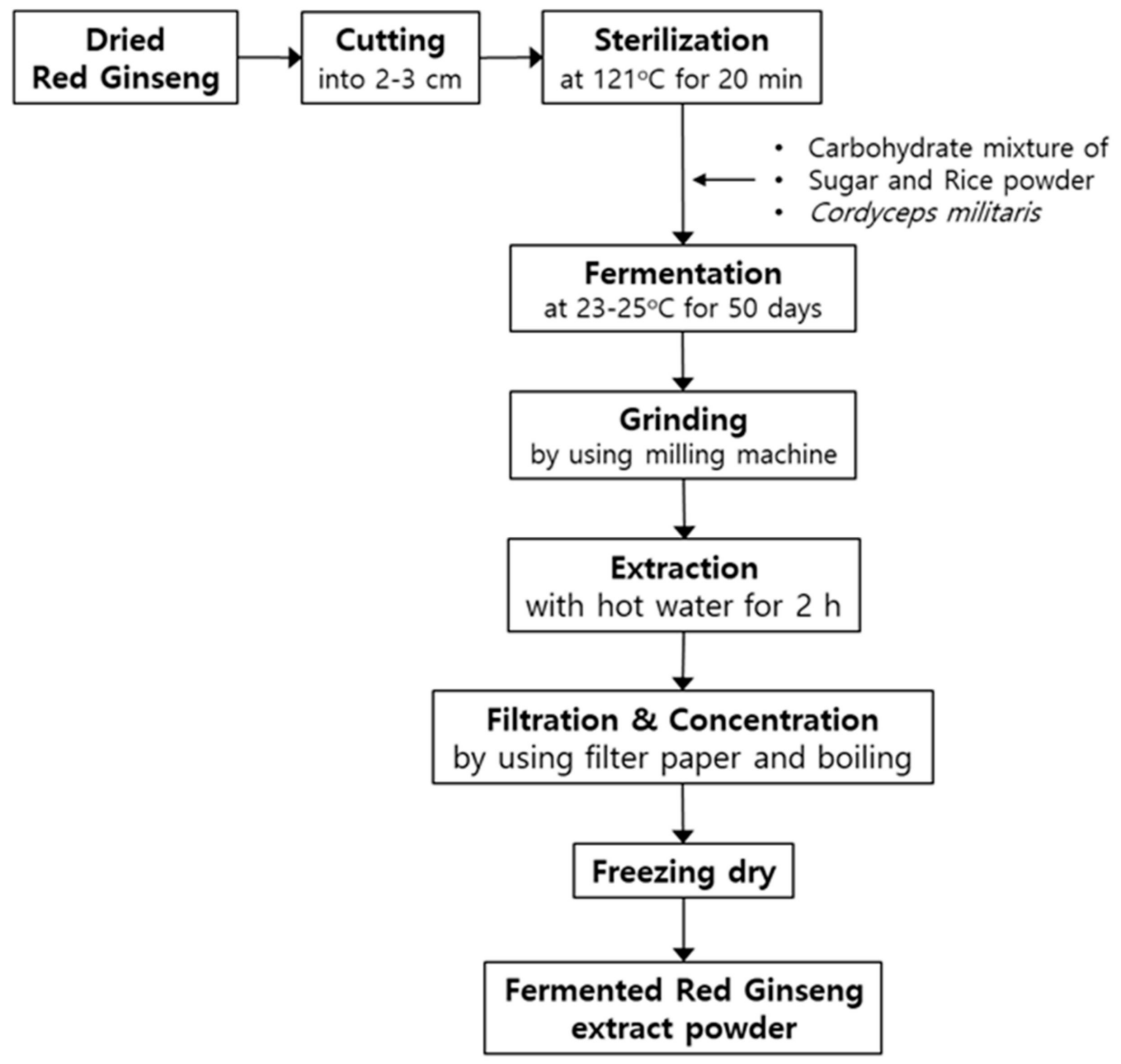
Extract preparation of solid-state fermented of Red Ginseng (RG) by *Cordyceps militaris*.

RG was provided by Glucan Inc. (Jinju, Korea). In the case of CRG, RG was fermented with *C. militaris* in a solid-state and extracted with hot water. Briefly, dried RG was cut into 2–3 cm long pieces and sterilized at 121 °C for 20 min. Thereafter, a carbohydrate mixture of sugar and rice powder with the same weight as the ginseng was added for settlement of mycelium to the surface of RG, and the cultured mycelium of *C. militaris* (Korea Culture Center of Microorganisms, 60304, Seoul, Korea) was mixed. The final mixture was incubated at 23–25 °C for 50 days. After incubation, the mycelium was covered to the surface of the RG by more than 90%. These were ground and extracted with hot water for 2 h. Hot water extract was then filtered through Advantec No. 2 filter paper (Advantec MFS Inc., Dublin, CA, USA), concentrated, freeze-dried, and then stored at 4 °C until used (Scheme 1). RG was prepared in a similar manner as the CRG, except for the fermentation process with *C. militaris* and the addition of carbohydrate mixture.

### 2.2. Content Analysis of Ginsenosides in CRG and RG

Content analyses of ginsenosides in CRG and RG were performed using the ultra-high-performance liquid chromatography (UPLC) system (AcquityTM UPLC system, Waters, Prague, Czech Republic) with a PDA detector and Acuity BEH C18 column (50 mm × 2.1 mm, 1.7 μm, Waters). Briefly, CRG and RG were dissolved in 70% methanol, filtered through a 0.2 μm syringe filter (Gelman Science, Ann Arbor, MI, USA), and separated using the UPLC system (Table 1).

The mobile phases were as follows: (I): distilled water (solvent A) and acetonitrile (solvent B) for ginsenoside Rg1, Re, Rf, Rh1, Rg2s, Rb1, Rc, Rb2, Rd, Rg3s, and Rg3r. The flow rate was 0.6 mL/min and the injection volume was 2 μL. The run time was 20 min. The samples were quantified by comparing the retention times to the authentic standards.

### 2.3. Animal Treatment

C57BL/6N male wild-type mice were obtained from Samtako Bio Korea (Osan, Korea). All animal experiments were approved by the Chungbuk National University Institutional Animal Care and Use Committee (IACUC). All animal experiments were performed under the IACUC guidelines and regulations. The animals were maintained in a specific-pathogen-free (SPF) facility under the following controlled conditions: temperature at 21 ± 2 °C, the humidity of 50% ± 10%, 12 h artificial light and dark cycles, and air exchange. High pressure was maintained in the experimental room to prevent contamination of the facility. C57BL/6N mice were fed with either a normal chow diet (NCD) or a fast-food diet (FFD, 40% calories from fat, 0.2% cholesterol, RD western diet, open source diets, plus fructose 23.1 g/L, and glucose 18.9 g/L added to the drinking water) (Table 2) for 14 weeks and treated with either 0.5% methylcellulose solution or CRG (100 or 300 mg/kg) once daily by oral gavage during the last 8 weeks.

### 2.4. Measurement of Serum Biochemistry

Blood samples were collected through a cardiac puncture. Serum was separated via centrifugation (7500 rpm, 15 min), and activity levels of alanine aminotransferase (ALT) and aspartate aminotransferase (AST) were analyzed by Green Cross Labcell (Yongin, Korea).

### 2.5. Primary Cell Culture

Mice were anesthetized with Zoletil (30 mg/kg; Virbac, Carros, France) and a catheter (24G) was inserted into the inferior vena cava (IVC). The liver was perfused with 30 mL of EGTA solution maintained at 37 °C using a Masterflex L/S easy-load II (Cole-Parmer Instrument Co, IL, USA). Thereafter, the liver was perfused with 75 mL of an enzyme buffer solution containing collagenase type 1 (650 µg/mL) (Worthington Biochemicals, LA, USA) and collagenase P (50 µg/mL) (Roche, Mannheim, Germany). The liver was dissected upon completion of the perfusion, minced well, and filtered through a 100 µm filter, and after two washes in the enzyme buffer solution, the hepatocytes were resuspended in complete M199 medium (Corning, NY, USA). To measure lipid accumulation, primary hepatocytes were incubated with palmitate (PA) (200 μM) with CRG and RG 62.5 μg/mL for 24 h for BODIPY staining. Also, for measuring the mRNA level, CRG and RG were treated at the same concentration 3 h before PA treatment, and then cultured for 6 h.

### 2.6. Cytotoxicity

Isolated primary hepatocytes were plated on collagen-coated 12-well (2 × 10^5^ cells/well) plates in filtered M199 medium supplemented with 10% fetal bovine serum (FBS) and 1% antibiotic antimycotic (Gibco, NY, USA). After 4 h, the medium was replaced with a fresh supplemented medium without fetal bovine serum and with the addition of CRG at a diverse concentration with or without palmitate (PA). After 24 h of incubation, the medium was collected for the required assays.

### 2.7. Quantitative Real-Time PCR Analysis

RNA extracted from the liver tissue was subjected to reverse transcription and subsequent PCR using a CFX connect real-time PCR system (Bio-Rad, Hercules, CA, USA). The targeted gene expression was normalized to β-actin expression levels as an internal control. The primer sequences are shown in Table 3.

### 2.8. Western Blot Assay

Liver tissues were directly homogenized for 30 min on ice with RIPA buffer. The lysates of the tissues were centrifuged at 13,000 rpm for 15 min. Protein concentration was measured using the Pierce BCA Protein Assay kit (Thermo Fisher Scientific Inc., Waltham, CA, USA). The supernatants of the lysates were separated by 12.5% SDS-PAGE and then were transferred to PVDF membranes. The membranes were blocked with 5% skim milk for 1 h at room temperature. Primary antibodies were diluted at 1:1000 and incubated overnight at 4 °C. The primary antibodies specific for Bcl-2 and horseradish peroxidase-conjugated secondary antibodies were used (Santa Cruz, CA, USA).

### 2.9. Histological Analysis

Liver tissues were removed from mice, then fixed with formalin and embedded in paraffin. To visualize lipid deposition, liver sections were stained using hematoxylin and eosin (H&E). Also, the frozen tissues were used for BODIPY staining. For quantitative analysis of lipid droplets, whole scanned liver sections were captured using a DMi8 (Leica, Wetzlar, Germany) at 200-fold magnification, and the BODIPY positive areas were measured using LAS X (Leica, Wetzlar, Germany) and ImageJ (NIH) software.

### 2.10. Statistical Tests

Statistical analyses were performed using the one-way ANOVA test on GraphPad Prism 7 (GraphPad Software Inc., San Diego, CA, USA). *p* values < 0.05 were considered statistically significant. Comparisons were made between the following groups for each study. * *p* < 0.0332 versus vehicle (Veh), ** *p* < 0.0021 versus Veh, *** *p* < 0.0002 versus Veh, **** *p* < 0.0001 versus Veh.

## 3. Results

### 3.1. CRG Treatment Prevents Hepatic Steatosis and Inflammation in FFD-Induced NAFLD

**Figure 1:**
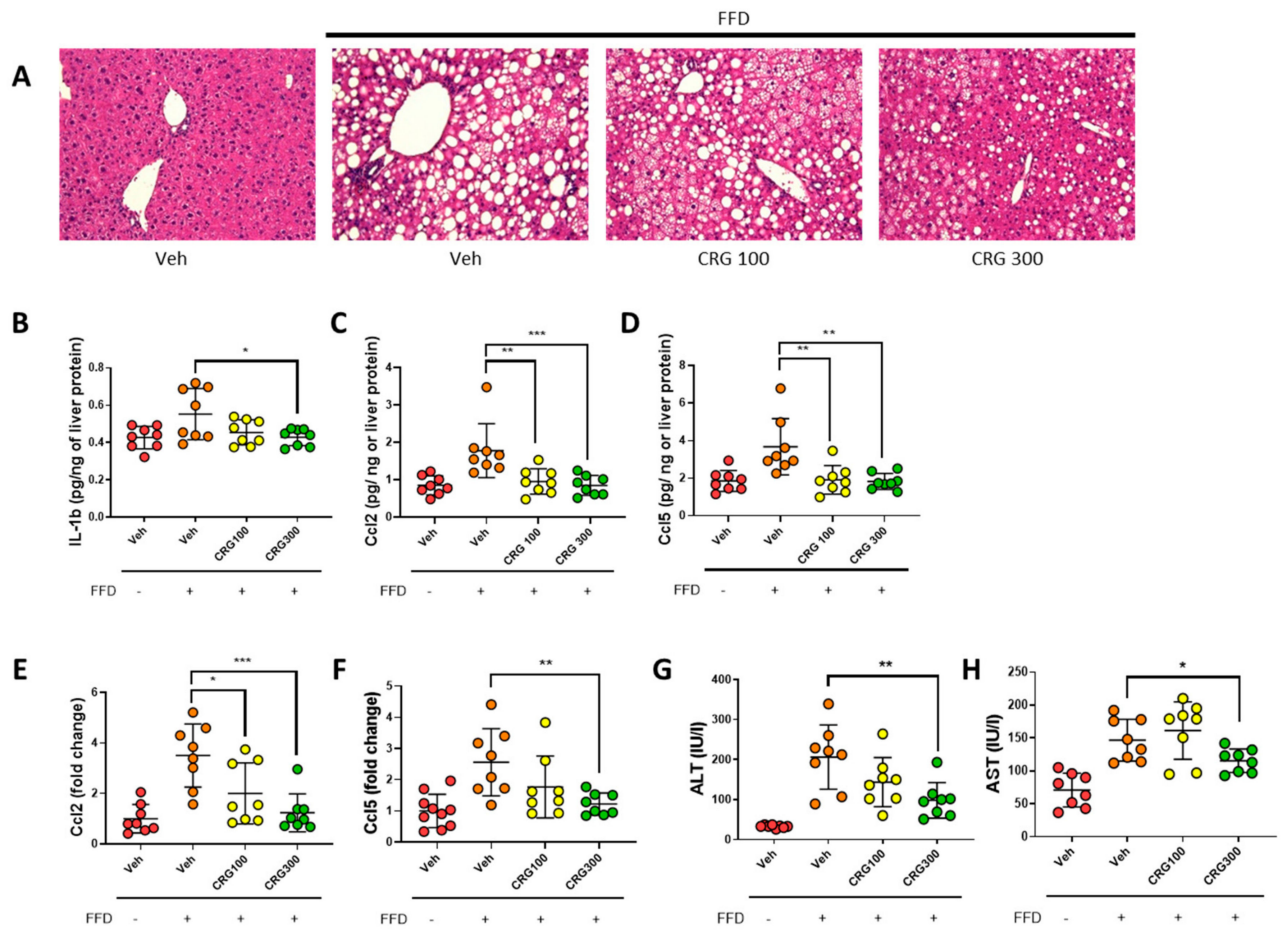
Fermented RG with *Cordyceps militaris* (CRG) treatment prevents inflammation and injury in fast-food diet (FFD)-induced non-alcoholic fatty liver disease (NAFLD). CRG was orally administered to mice for the last 8 weeks till the animals were sacrificed. (**A**) Each section was stained with H&E. The protein levels of (**B**) Il-1β; (**C**) Ccl2 and (**D**) Ccl5 in liver tissue lysates were determined by ELISA. Expressions of (**E**) Ccl2 and (**F**) Ccl5 in liver tissue were measured by qRT-PCR shown as fold change compared with normal chow diet (NCD) mice. The level of (**G**) alanine aminotransferase (ALT) and (**H**) aspartate aminotransferase (AST) in the serum were measured. Original magnification, X 20. * *p* < 0.05 versus vehicle (Veh), ** *p* < 0.01 versus Veh, *** *p* < 0.001 versus Veh.

To investigate the effect of CRG in NAFLD, mice were subjected to an FFD diet that closely mimics most aspects of human NASH [38]. Histological analysis revealed that infiltration of inflammation and lipid accumulation were reduced with the treatment of CRG in a dose-dependent manner in mouse liver (Figure 1A). Consistently, CRG decreased FFD-induced hepatic inflammation including Il-1β, Ccl2, and Ccl5 (Figure 1B–F). Furthermore, the treatment of CRG protected against FFD-induced liver injury as demonstrated by the reduction of serum level of ALT and AST (Figure 1G,H).

**Figure 2:**
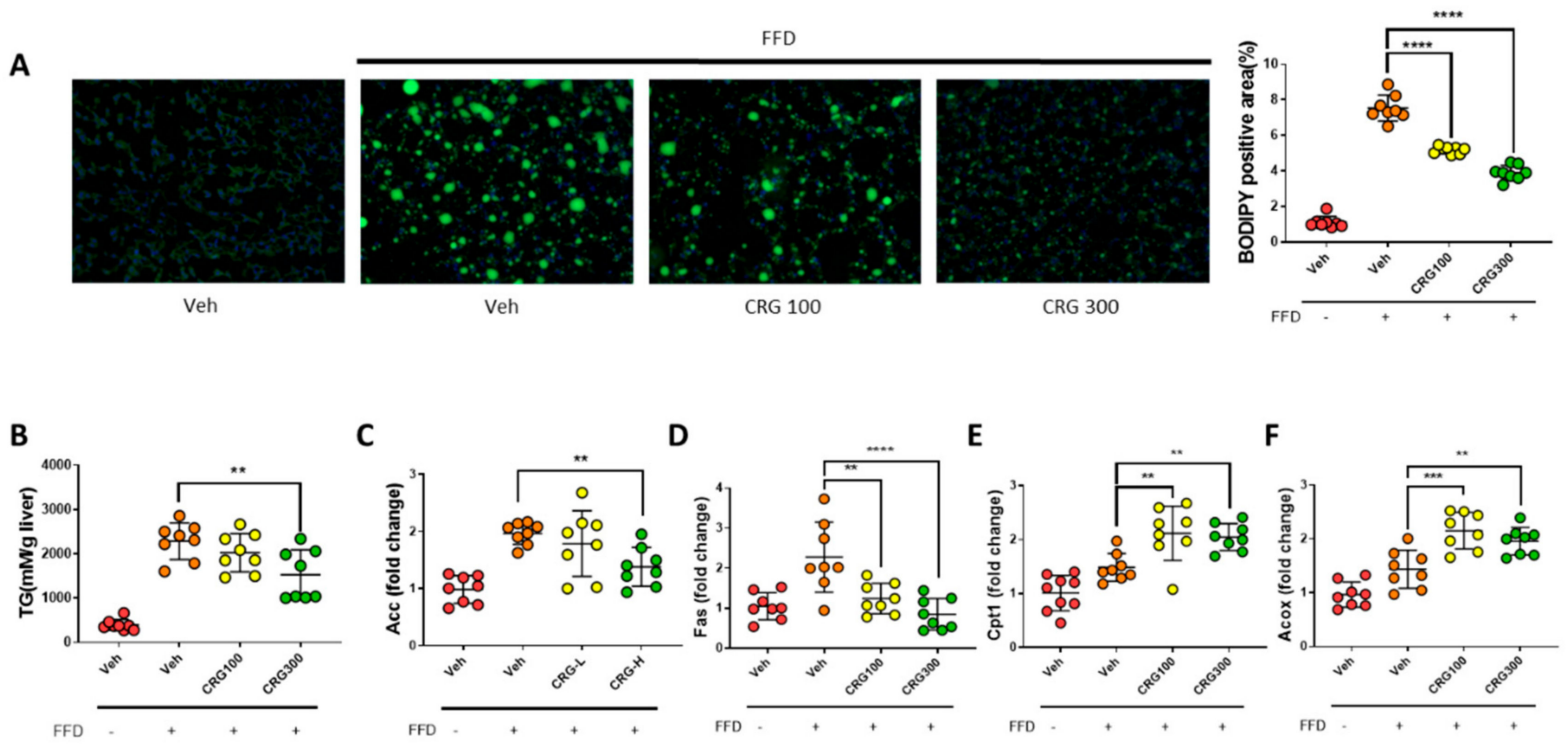
CRG treatment prevents hepatic steatosis in FFD-induced NAFLD. (**A**) Each section was stained with BODIPY to determine lipid accumulation. (**B**) Hepatic triglyceride (TG) level was measured. Expression of fatty acid synthesis genes such as (**C**) Acc and (**D**) Fas; and fatty acid oxidation genes (**E**) Cpt1 and (**F**) Acox were evaluated by qRT-PCR shown as fold change compared with NCD diet mice. Original magnification, X 20. ** *p* < 0.01 versus vehicle (Veh), *** *p* < 0.001 versus Veh, **** *p* < 0.0001 versus Veh.

To determine the effect of CRG on liver steatosis, FFD-induced lipid droplets were evaluated by the quantification of the BODIPY-positive area. As shown in Figure 2A, hepatic lipid accumulation was markedly decreased in high doses of CRG-treated mouse liver. In accordance with the results of the BODIPY staining, the levels of hepatic triglyceride (TG) were significantly decreased in the CRG-treated group (Figure 2B). Moreover, the administration of CRG significantly reduced the FFD-induced hepatic expressions of lipogenic genes such as Acc and Fas. Also, CRG administration induced the hepatic expressions of fatty acid oxidation such as Cpt1, Acox1 (Figure 2C–F). These data indicate that CRG treatment ameliorates hepatic steatosis, liver injury, and inflammation, and suggest the extract of CRG contains protective compounds against the pathogenesis of NAFLD.

### 3.2. Fermentation of RG with C. militaris Enhances the Protective Effect on Lipid Accumulation and Inflammation

**Figure 3:**
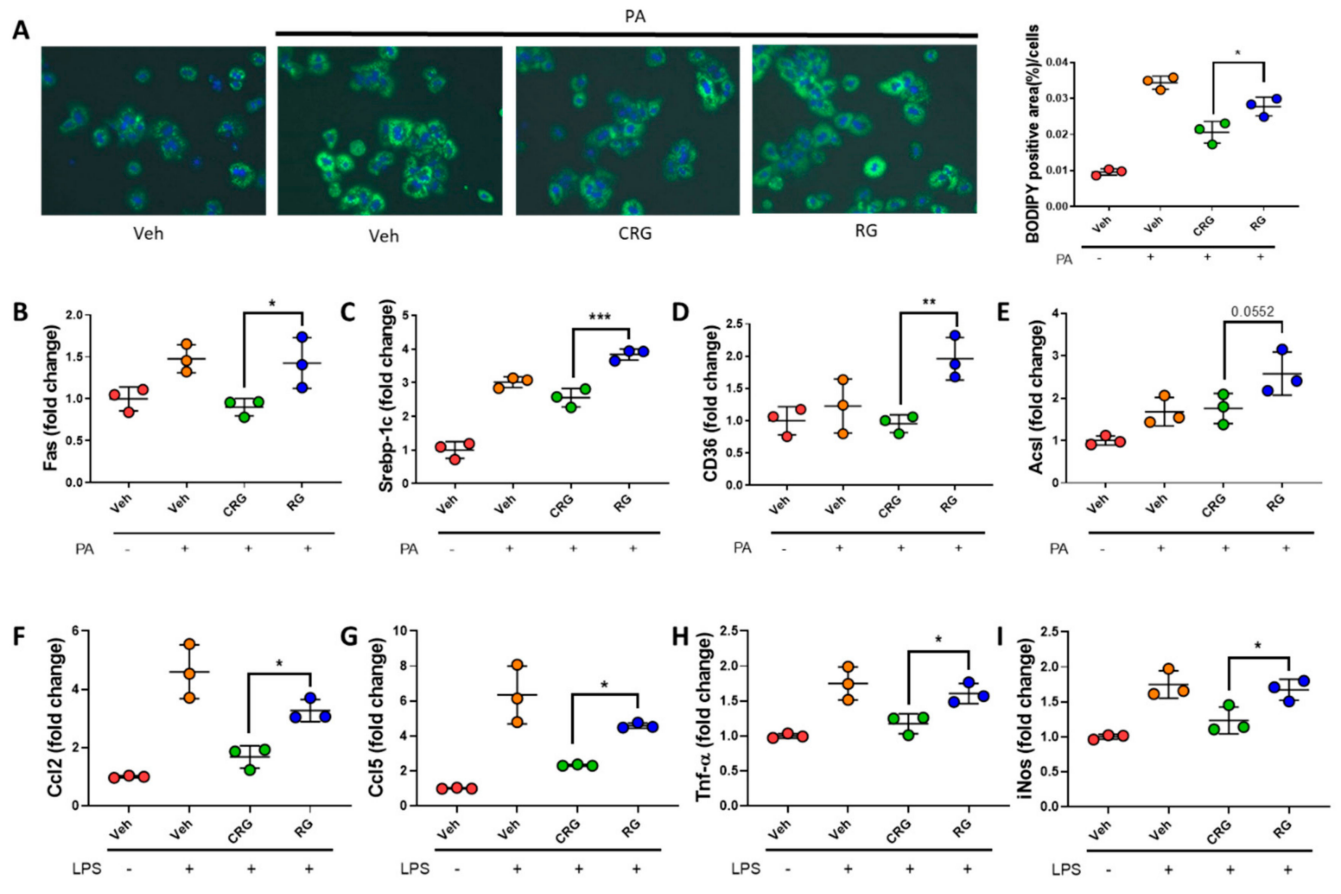
Fermentation of RG with *C. militaris* enhances the protective effect on lipid accumulation and inflammation. (**A**) Lipid accumulation was assessed by BODIPY staining. Expression of fatty acid synthesis genes (**B**) Fas; (**C**) Srebp-1c and fatty acid oxidation genes marker (**D**) CD36; and (**E**) Acsl determined by qRT-PCR shown as fold change compared with Bovine serum albumin (BSA). The expression of inflammatory genes (**F**) Ccl2; (**G**) Ccl5; (**H**) Tnf-α and (**I**) iNos was measured by qRT-PCR. Original magnification, X 20. * *p* < 0.05 versus vehicle (Veh), ** *p* < 0.01 versus Veh, *** *p* < 0.001 versus Veh.

To compare the effect of CRG or RG on steatosis, we measured PA-induced lipid accumulation in the primary hepatocytes. The cytotoxicity test was performed for the set concentration of CRG (Figure S1). Both CRG and RG treatments significantly decreased lipid accumulation compared with the PA treatment (Figure 3A). Based on these data, we screened various genes that are closely related to lipid metabolism according to CRG and RG treatments. Among them, we found the expressions of Fas, Srebp-1c, CD36, and Acsl are significantly changed (Figure 3B–E and Figure S3). CRG-mediated decrease lipogenesis and lipid uptake were greater than that for conventional RG. Moreover, the anti-inflammatory activities of CRG were significantly stronger than those of conventional RG (Figure 3F–I). Collectively, these data show that fermented RG with *C. militaris* has a superior protective effect than conventional RG on lipid metabolism and inflammation.

### 3.3. CRG has High Contents of Ginsenoside Rd and Rg3 that Inhibit Lipid Accumulation

**Figure 4:**
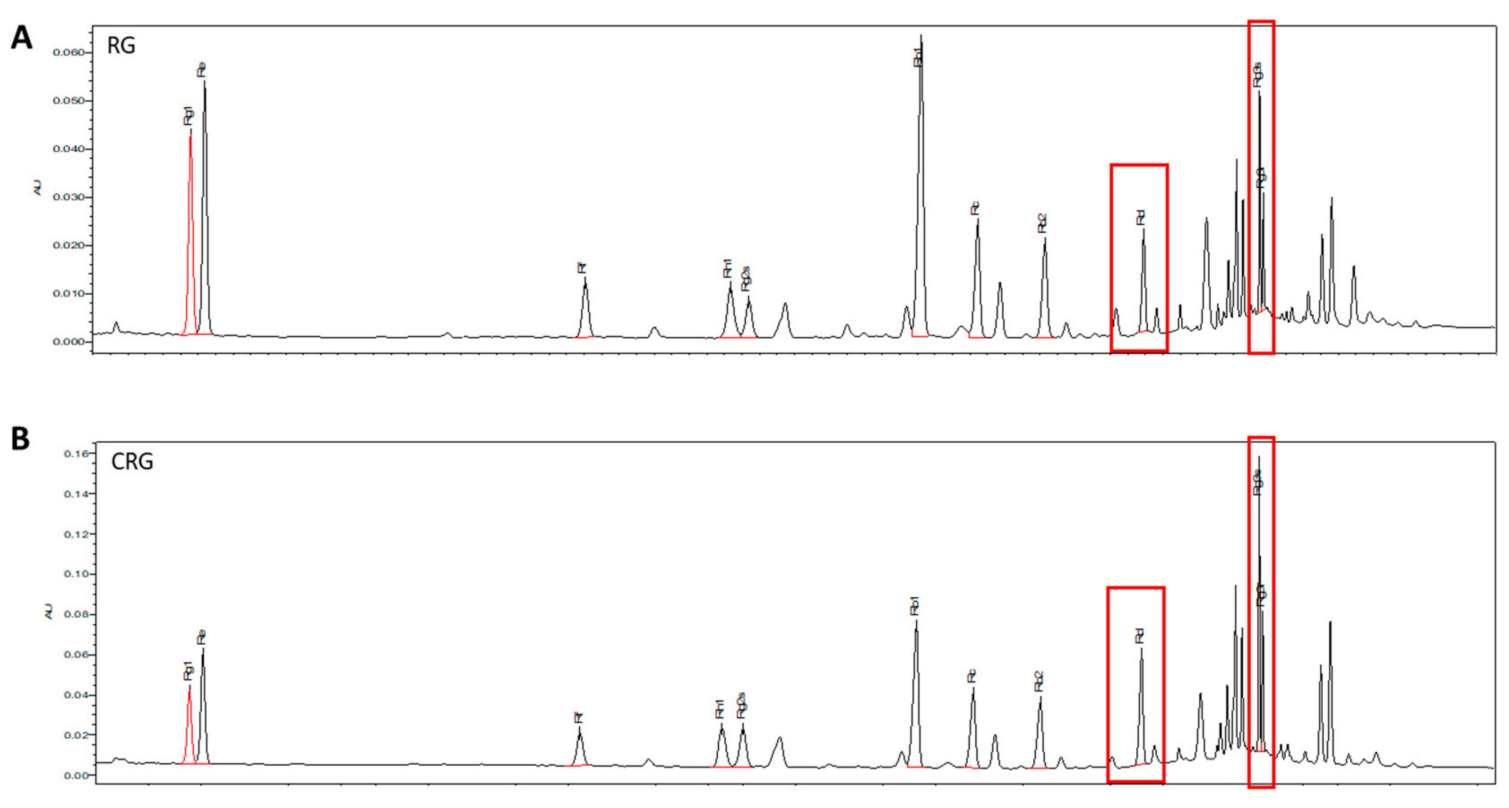
Ultra-high-performance liquid chromatography (UPLC) chromatogram of ginsenosides in (**A**) RG and (**B**) CRG. The first red box is Rd, the second one is Rg3.

**Figure 5:**
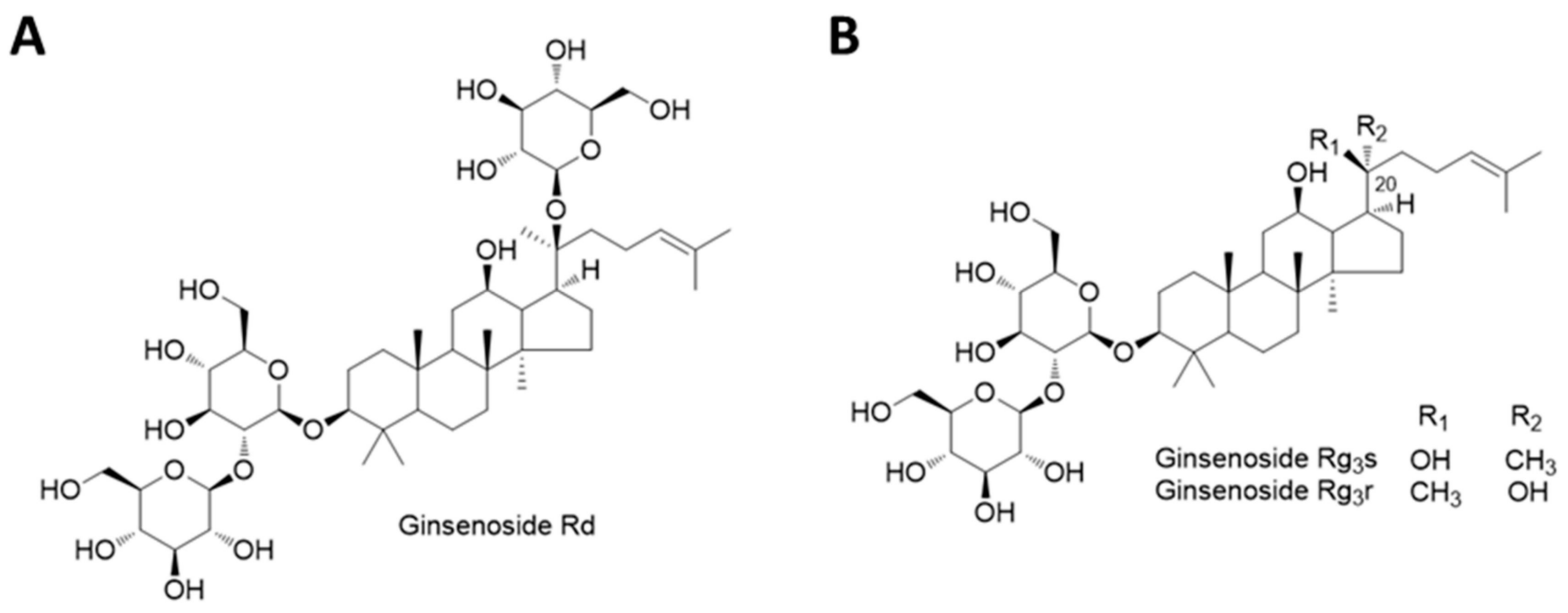
The chemical structure of (**A**) Rd; (**B**) Rg3s, and Rg3r.

The results of the superior effect of CRG (Figure 3) prompted us to test whether CRG contains more functional compounds. Thus, we analyzed the extract of CRG and RG by UPLC (Figure 4). As shown in Table 4, the CRG extract has significantly increased amounts of some ginsenosides (Rf, Rd, Rg3s, and Rg3r), especially Rd and Rg3 (Figure 5), compared with the RG extract.

### 3.4. Rd and Rg3 Protects PA-Induced Aberrant Activation of mTORC1 and Subsequent Inhibition of Mitophagy and PPARα

**Figure 6:**
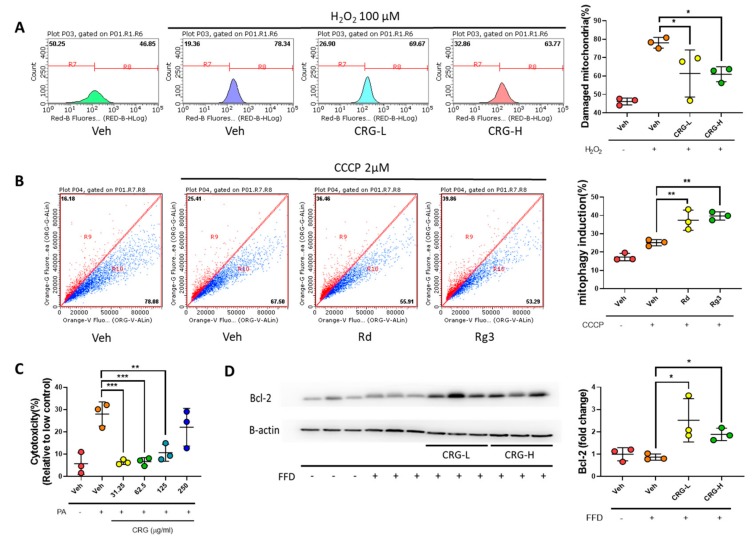
Rd and Rg3 protect mitochondria-mediated lipotoxic apoptosis. (**A**) Hep3B cells were measured for MitoSOX 12 h after treatment with H_2_O_2_ and CRG. (**B**) Hep3B cells were transfected with pLVX-puro-mt-Keima. Analysis of expressing mt-Keima treated with ginsenoside Rd for 24 h. Rd mediated mitophagy was quantified by flow cytometry with 2 μM CCCP. The excitation wavelength is 586 nm in neutral and 440 nm in acidic environment. (**C**) PA-induced cytotoxicity was measured by LDH. (**D**) Representative western blotting analysis of Bcl-2. CRG 62.5 μg/mL is referred to as CRG-H and CRG 31.25 μg/mL as CRG-L. * *p* < 0.05 versus vehicle (Veh), ** *p* < 0.01 versus Veh, *** *p* < 0.001 versus Veh.

Because mitochondria play an important role in lipid metabolism, we measured mitochondrial superoxide (MitoSOX) to determine the effect of CRG on mitochondria (Figure 6A). As a result, the CRG reduced mitochondrial damage. Given that maintaining mitochondrial integrity is critical for lipid-mediated pathophysiology in the liver [39], we analyzed the effect of Rd and Rg3 on mitophagy as major mechanisms of mitochondrial quality control [40]. Using the Mt-Keima system [41], Rd and Rg3 increased mitophagy in response to Carbonyl cyanide m-chlorophenylhydrazone (CCCP) treatment compared with the control group (Figure 6B), indicating that these ginsenosides improve mitochondrial integrity under the stress. Next, we evaluated PA-induced cytotoxicity in primary hepatocyte. Indeed, CRG protected cell death against PA (Figure 6C). Because mitophagy has been reported to confer cellular protection through the induction of anti-apoptotic Bcl-2 expression [42], we analyzed the hepatic expression of Bcl-2. Bcl-2 was decreased in the liver of the FFD group and this reduction was recovered after CRG treatment (Figure 6D). Taking together, our data suggest that CRG induces mitophagy and Bcl-2 expression which inhibit lipotoxic apoptosis in NAFLD.

**Figure 7:**
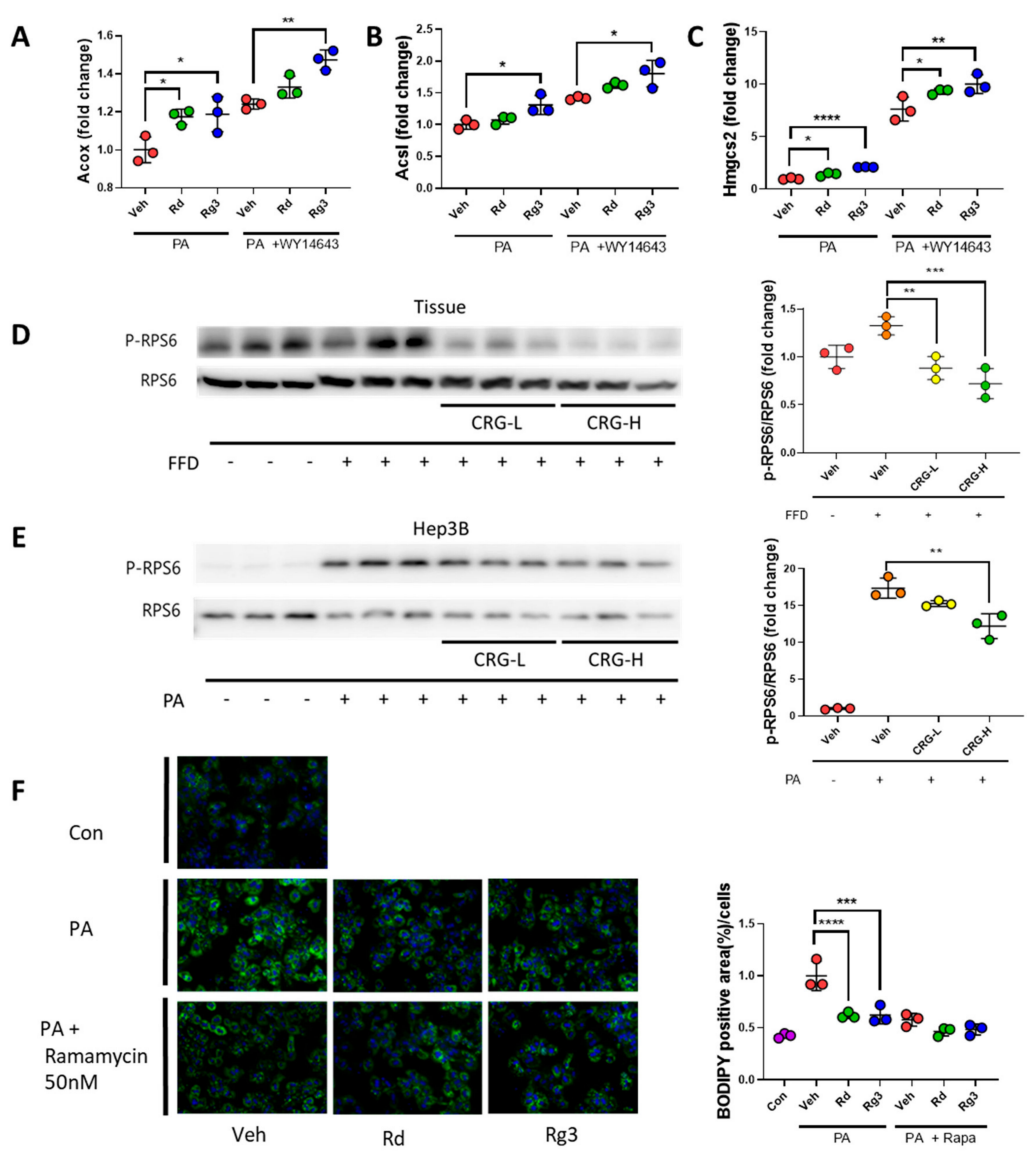
Rd and Rg3 -induced anti-steatotic effect is dependent on mTORC1. (**A**–**C**) Primary hepatocytes were incubated with PA (125 μM) and WY14643 (30 μM) for 6 h. The expression of PPARα target genes (**A**) Acox; (**B**) Acsl and (**C**) Hmgcs2 were determined by qRT-PCR. (**D**) The liver tissue and (**E**) Hep3b cells were incubated with PA (200 μM) and CRG for 24 h. (**F**) Primary hepatocytes were incubated with PA (200 μM) and Rapamycin (50 nM) with 2.5 μg/mL Rd or 5 μM Rg3 for 24 h. Lipid accumulation was assessed by BODIPY staining. Original magnification, X 20. * *p* < 0.05 versus vehicle (Veh), ** *p* < 0.01 versus Veh, *** *p* < 0.001 versus Veh, **** *p* < 0.0001 versus Veh.

To clarify the effect of Rd and Rg3 on mitochondrial function, we next examined the PPARα-mediated β-oxidation which mainly occurs in mitochondria [43]. Indeed, the treatment of Rd and Rg3 increased the β-oxidation-related genes expressions such as Acox, Acsl, and Hmgcs2 (Figure 7A–C). Of note, these ginsenosides-mediated increments were further amplified in response to WY14643, a PPARα activator. mTORC1 has been known to regulate both mitophagy and PPARα signaling [44]. We then investigated the effect of Rd and Rg3 on mTORC1 signaling pathway in NAFLD. We found PA itself significantly induced mTORC1 activation, as demonstrated by phosphorylation of RPS6, and this activation was inhibited by CRG in a dose-dependent manner (Figure 7D,E). To rule out the involvement of PA-induced cell death for changes in mTORC1, we investigated the effects of Rd and Rg3 on mTORC1 induced by physiological stimulation of mTORC1 with FBS. Consistent with PA-induced model, ginsenosides inhibited the FBS-induced activation of mTORC1 (Figure S2). Next, we determined whether the anti-steatotic effects of Rd and Rg3 are mediated by mTORC1 signaling, we performed BODIPY staining on primary hepatocyte with rapamycin, which is a canonical inhibitor of the mTORC1 serine/threonine kinase. As expected, the level of lipid accumulation was significantly decreased with Rd and Rg3 treatments in the PA-induced primary hepatocytes. However, these effects were abrogated in the condition of rapamycin treatment (Figure 7F). Taken together, our data suggest that Rg3 and Rd, which are abundant in CRG, confer protection against steatosis through mTORC1-mediated induction of mitophagy and PPARα signaling.

### 3.5. Rd and Rg3 Exerts Anti-Inflammatory Activity via mTORC1-Mediated M2 Polarization

**Figure 8:**
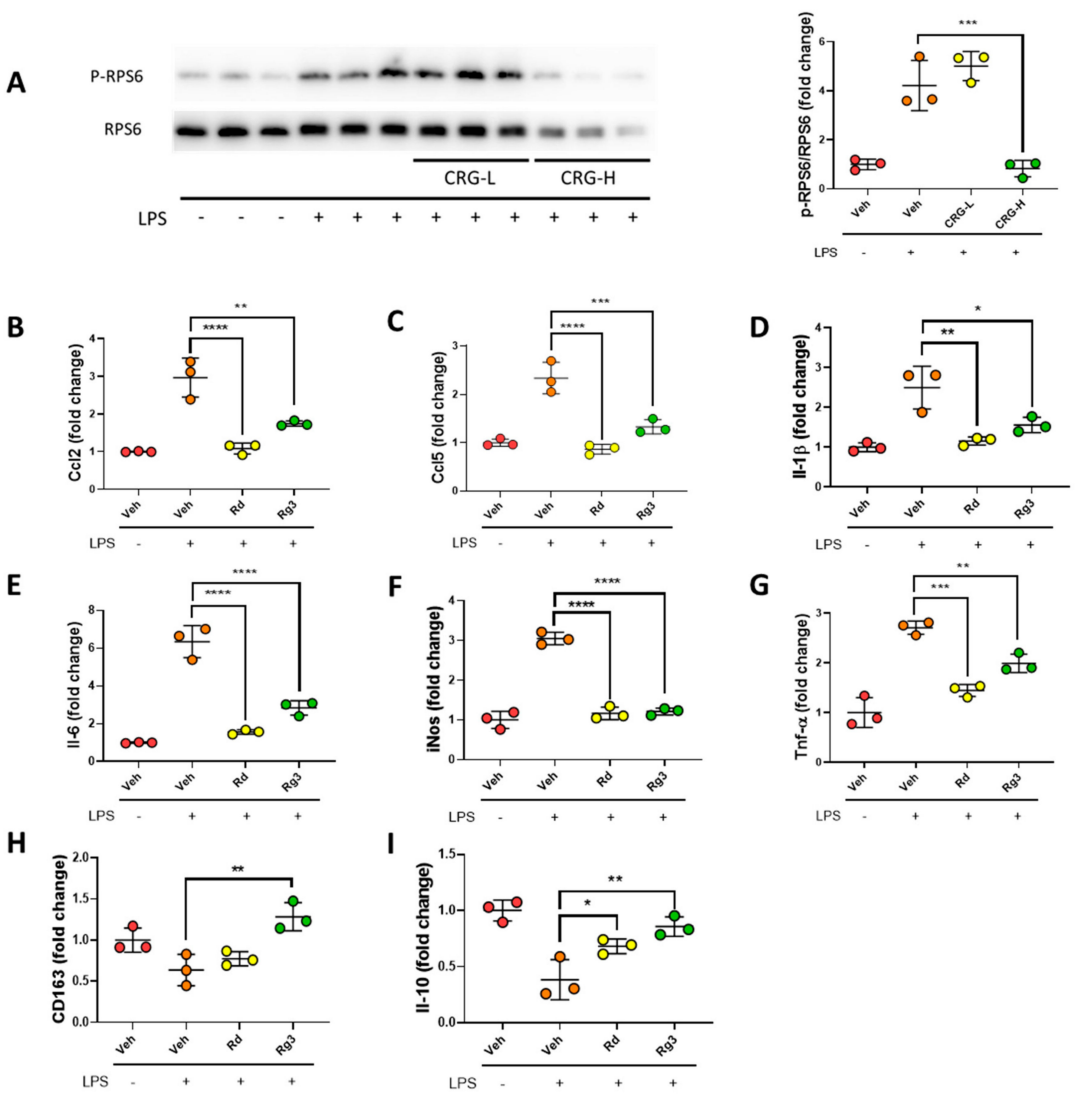
Rd and Rg3 exert anti-inflammatory activity via mTORC1-mediated M2 polarization. (**A**) Raw264.7 cells were incubated with LPS and CRG for 24 h. (**B**–**I**) RAW264.7 cells were pretreated with Rd or Rg3 for 3 h and then stimulated with LPS for 6 h. Expression of M1 marker genes (**B**) Ccl2; (**C**) Ccl5; (**D**) Il-1β; (**E**) Il-6; (**F**) iNos and (**G**) Tnf-α and (**C**) M2 marker genes (**H**) CD163 and (**I**) Il-10 were measured by qRT-PCR. * *p* < 0.05 versus vehicle (Veh), ** *p* < 0.01 versus Veh, *** *p* < 0.001 versus Veh, **** *p* < 0.0001 versus Veh.

Because CRG inhibited liver injury and inflammation (Figure 1), we hypothesized CRG has protective factors in the inflammatory process. To determine the anti-inflammatory potential of Rd and Rg3, mRNA expressions of various inflammatory genes were measured in macrophages. In accordance with in vivo data (Figure 1), the treatment of CRG decreased LPS-induced expression of pro-inflammatory cytokines (Figure 3F–I). Interestingly, CRG decreased LPS-induced activation of mTORC1 (Figure 8A). Because mTORC1 activation has been known to induce the M1 polarization [45], we determined the effect of these ginsenosides on the polarization of macrophages. Treatment with Rd and Rg3 decreased the marker for M1 such as Ccl2, Ccl5, Il-1β, Il-6, iNos, and TNF-α, while the marker for M2 (CD163 and Il-10) increased in the inflammatory response induced by LPS (Figure 8B–I). These data suggest that CRG extract enriched in Rd and Rg3 induces the level of polarization toward M2 through the regulation of mTORC1 activity.

## 4. Discussion

RG is a natural medicine used in Asian countries for a long time and has a therapeutic effect on various diseases. Especially, recent studies have shown that Korean RG is also effective in alleviating liver diseases [46,47,48]. In our study, CRG has a better protective effect than RG against lipid metabolism and inflammation. Especially, Rd and Rg3 showed the induction of mitophagy and PPARα. In addition, the treatment of Rd and Rg3 induced M2 polarization in macrophages and showed anti-inflammatory responses. The changes in mitophagy and M1/M2 polarization both depend on mTORC1 signaling [16,45]. Consequently, the administration of CRG inhibits hepatic steatosis and inflammation in the FFD-induced NAFLD model.

Several lines of evidence support that the treatment of CRG mitigates NAFLD. First, the hepatoprotective and fat accumulation inhibitory effects of ginsenosides are well known in various liver disease models [49,50,51]. Second, CRG and ginsenosides show anti-inflammatory responses [52,53]. Third, mitophagy, a mitochondrial quality control, is known to influence the regulation of lipid metabolism in the liver, and mitophagy increased during CRG treatment [54]. Finally, mTORC1 inhibition by CRG improves NAFLD in vivo and activates PPARα to reduce fat accumulation, followed by M2 polarization in Raw264.7 cells in vitro. Taken together, our results suggest that the extract of CRG alleviates NAFLD through mTORC1 inhibition-mediated mitophagy induction and M2 polarization.

For clarification of events downstream of mTORC1, we treated with rapamycin, a well-known inhibitor of mTORC1, in hepatocytes. In contrast to the reduction of fat accumulated by PA when Rd and Rg3 alone were treated, treatment of Rd and Rg3 with Rapamycin did not change lipid droplets significantly. However, lipid accumulation decreased in PA + Rapa group, similarly to the control group, and there is a limit to the correlation between mTORC1 and Rd/Rg3. In addition, β-oxidation was amplified by ginsenoside treatment more than WY14643, an activator of PPARα which is a downstream signal of mTORC1 [55,56]. Therefore, we speculate that CRG has a therapeutic effect on NAFLD via the mTORC1 pathway. This concept corresponds to the mTORC1 signaling pathway and shows significant decreases in fat and inflammatory responses in mice administered with CRG.

To determine the effect of CRG on mitochondrial function, we measured mitophagy using the mt-Keima system. More mitophagy was induced in Rd and Rg3-treated groups than in CCCP-treated only groups. Recent studies have shown that ER stress induced by lipid toxicity aggravates NAFLD and prevents proper autophagy [57,58]. Autophagy also participates in the regulation of apoptosis and affects cell survival [42,59,60]. Among autophagy, mitophagy is known to inhibit the production of ROS which induces apoptosis [61,62,63]. In the present study, CRG not only reduced cytotoxicity due to lipid toxicity but also increased the expression of Bcl-2. These data suggest that increased mitophagy with CRG treatment mitigates NAFLD by inducing Bcl-2 expression, which inhibits apoptosis by lipotoxicity.

To elucidate the effects of Rd and Rg3 on anti-inflammatory responses due to polarization on macrophage, we treated these ginsenosides with LPS in RAW264.7 cells. The increase of mTORC1 with LPS treatment in RAW264.7 cells was decreased during Rd and Rg3 treatment and also showed anti-inflammatory response through decreased M1 markers and increased M2 markers. Since the association between mTORC1 and M1/M2 polarization in macrophages is already known [45], these results suggest that CRG alleviates NAFLD through mTORC1 inhibition-mediated M2 polarization.

## 5. Conclusions

In conclusion, CRG enriched in Rd and Rg3 is effective for the prevention of NAFLD because its inhibition of mTORC1 signaling confers anti-steatotic function through induction of mitophagy and anti-inflammatory activity via M2 polarization, respectively.

## Figures and Tables

**Table 1:** Protocol for LC-MS of Korean Red Ginseng fermented with *Cordyceps militaris* extract.

LS-MS Condition	
Column	ACQUITY BEH C18 (50 mm x 2.1 mm, 1.7μm)
Flow rate	0.6 mL/min
Injection volume	2 μL
UV Absorbance	203 nm
Column temperature	40 ℃

**Table 2:** Composition of the fast-food diet.

Component	Western Diet (g)	Sugar Solution (g/L)
Casein, Lactic, 30 Mesh	195	0
Methionine, DL	3	0
Sucrose, Fine Granulated	350	0
Lodex 10	100	0
Starch, Corn	50	0
Solka Floc, FCC200	50	0
Butter, Anhydrous	200	0
Corn Oil	10	0
S10001A	17.5	0
Calcium Phosphate, Dibasic	4	0
Choline Bitartrate	2	0
V10001C	1	0
Ethoxyquin	0.04	0
Cholesterol, NF	1.5	0
Sucrose	0	23.1
Glucose	0	18.9
Total	1001.54	42

**Table 3:** Primer sequences of genes used for quantitative real time- polymerase chain reaction (q-RT PCR).

Gene	Forward (5′ to 3′)	Reverse (5′ to 3′)
Acc	GGACAACACCTGTGTGGTAGAA	CGTGGGGATGTTCCCTCT
Fas	AGGTGCTAGAGGCCCTGCTA	GTGCACAGACACCTTCCCAT
Srebp-1c	GGAGCCATGGATTGCACATT	AGGAAGGCTTCCAGAGAGGA
Cpt-1	CGGTTCAAGAATGGCATCATC	TCACACCCACCACCACGAT
Acox1	CTCACTCGAAGCCAGCGTTA	TTGAGGCCAACAGGTTCCAC
CD36	AATGAGACTGGGACCATCG	CTCCAACACCAAGTAAGACCAT
Acsl	ACCATCAGTGGTACCCGCTA	CGCTCACCACCTTCTGGTAT
Hmgcs2	ATACCACCAACGCCTGTTATG	CAATGTCACCACAGACCACCA
Ccl2	ATTGGGATCATCTTGCTGGT	CCTGCTGTTCACAGTTGCC
Ccl5	ACTCCCTGCTGCTTTGCCTAC	TGTATTCTTGAACCCACTTCTTCTCTG
Tnf-α	AGGGTCTGGGCCATAGAACT	CCACCACGCTCTTCTGTCTA
Il-1β	CTCGCAGCAGCACATCAACAAG	CCACGGGAAAGACACAGGTAGC
Il-6	ACAAAGCCAGAGTCCTTCAGAGAG	TTGGATGGTCTTGGTCCTTAGCC
CD163	TCCACACGTCCAGAACAGTC	CCTTGGAAACAGAGACAGGC
Il-10	GCTGGACAACATACTGCTAACCG	TCCGATAAGGCTTGGCAACCC
iNos	GCACCACCCTCCTCGTTCAG	TCCACAACTCGCTCCAAGATTCC
β-actin	CATCCGTAAAGACCTCTATGCCAAC	ATGGAGCCACCGATCCACA

**Table 4:** Content of eleven ginsenosides in RG and CRG. * *p* < 0.05 versus RG.

Sample	Rg1	Re	Rf	Rg2s	Rb1	Rc	Rb2	Rd	Rg3s	Rg3r	Rk1
RG	2.38 ± 0.14	3.13 ± 0.37	0.93 ± 0.05	1.12 ± 0.55	6.06 ± 0.21	2.49 ± 0.03	1.69 ± 0.13	1.06 ± 0.01	1.50 ± 0.80	0.79 ± 0.45	0.62 ± 0.08
CRG	1.05 ± 0.44	1.63 ± 0.74	1.32 ± 0.09 *	2.31 ± 0.35	6.82 ± 0.54	2.98 ± 0.44	2.07 ± 0.33	2.23 ± 0.28 *	3.50 ± 0.29 *	2.04 ± 0.33 *	1.36 ± 0.14 *

## Supplementary Materials

The following are available online at https://www.mdpi.com/2072-6643/11/12/2963/s1, Figure S1: CRG has no cytotoxic effect at low concentrations, Figure S2: Rd and Rg3 reduce mTORC1 activation induced by FBS stimulation, Figure S3: CRG treatment prevents lipid accumulation.
