# Supplementary figures and images for: Fermented Korean Red Ginseng Extract Enriched in Rd and Rg3 Protects against Non-Alcoholic Fatty Liver Disease through Regulation of mTORC1
Source: Nutrients. 2019 Dec 4;11(12):2963. doi: 10.3390/nu11122963 (PMC6949916; doi:10.3390/nu11122963)

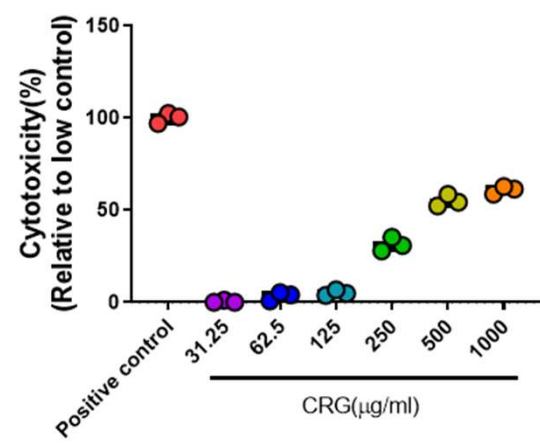

Supplement: Supplementary file 1 [file nutrients-11-02963-s001.zip › Fig. S1.pdf]

**A**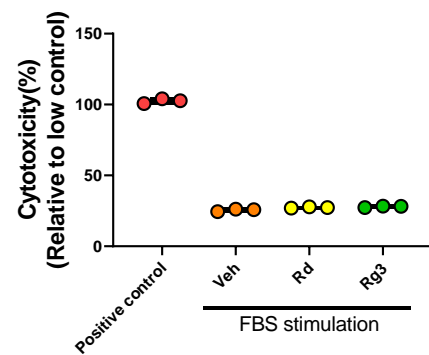**B**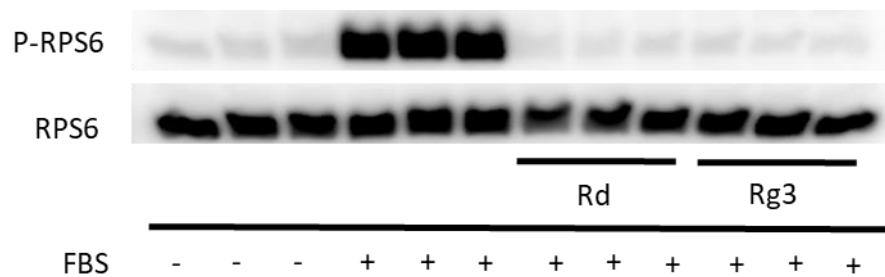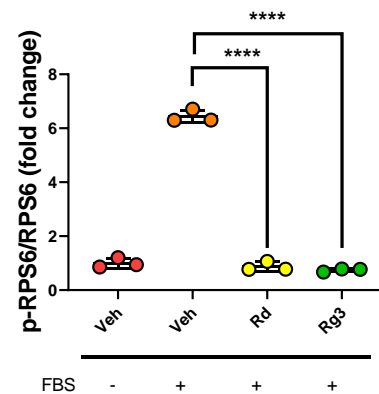

Supplement: Supplementary file 1 [file nutrients-11-02963-s001.zip › Fig. S2.pdf]

A

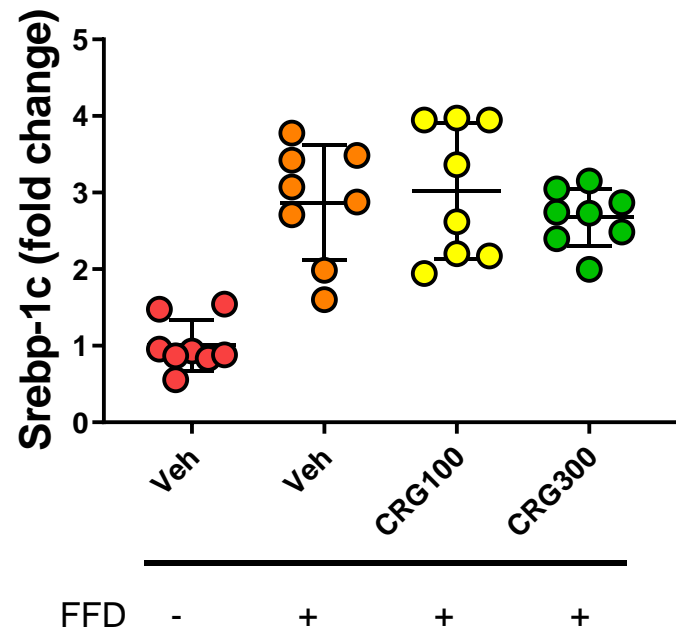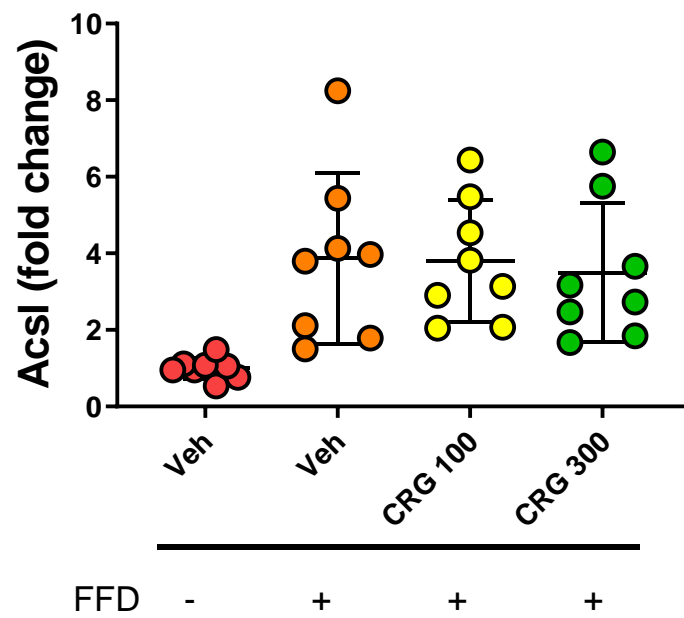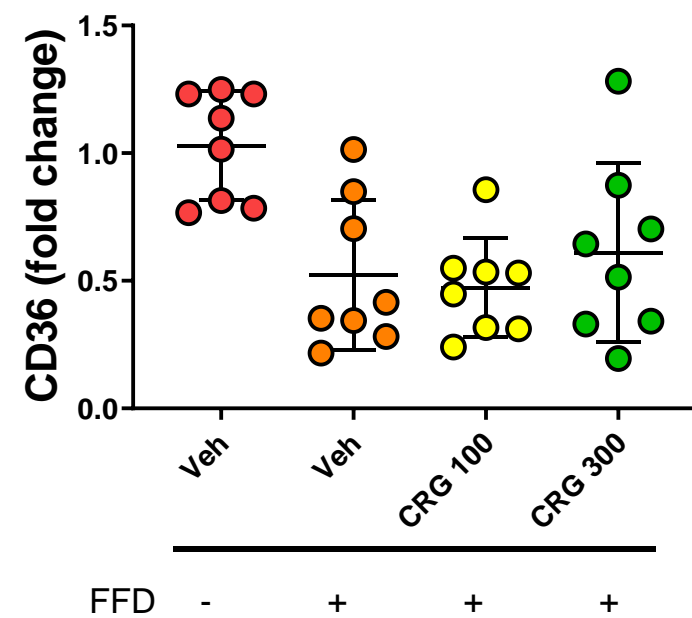

B

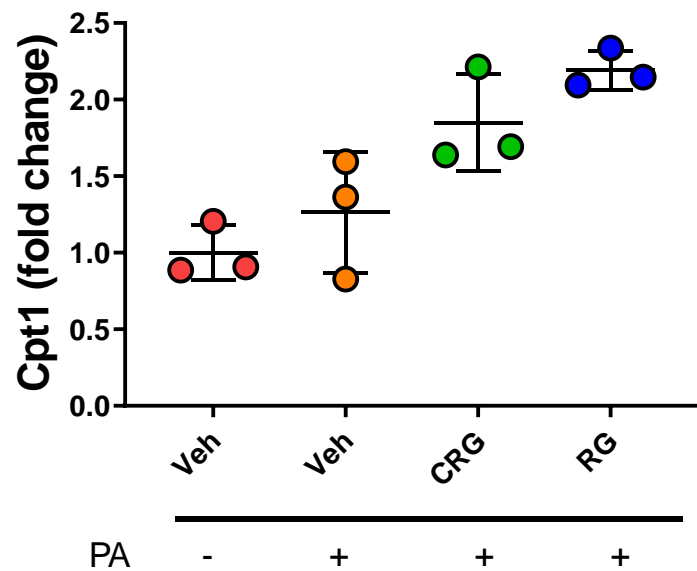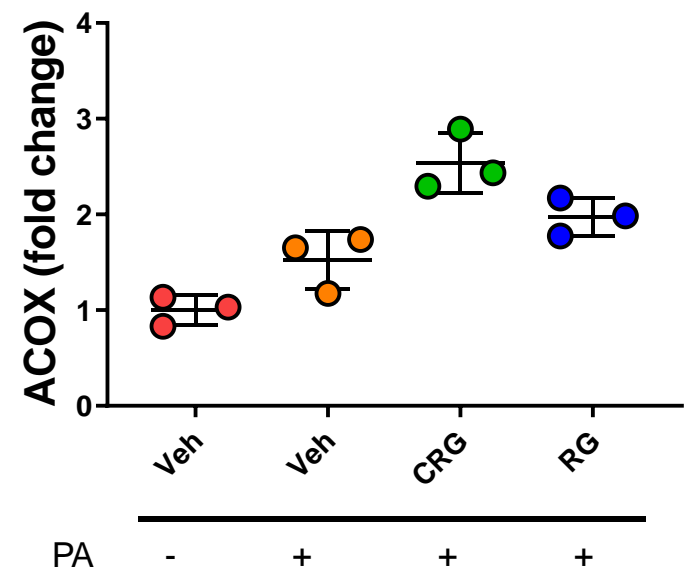

Supplement: Supplementary file 1 [file nutrients-11-02963-s001.zip › Fig. S3.pdf]
